# Incidence of stroke in patients with atrial fibrillation undergoing surgical treatment: a meta-analysis

**DOI:** 10.1186/s12872-025-04605-y

**Published:** 2025-03-29

**Authors:** Deqing Lin, Yongbo Cheng, Sanjiu Yu, Xin Liu, Chaojun Yan, Wei Cheng

**Affiliations:** Department of Cardiac Surgery, Southwest Hospital, Third Military Medical University (Army Medical University), Chongqing, 400038 P.R. China

**Keywords:** Atrial fibrillation, Stroke, Surgery, Coronary artery bypass, Meta-analysis

## Abstract

**Introduction:**

Atrial fibrillation (AF) is self-limiting condition, but it may also increase the risk of stroke and death. The association between AF and surgery with stroke was assessed both subjectively and statistically using systematic review and meta-analysis.

**Methods:**

For data collection, a thorough search was made in PubMed, EMBASE, Science Direct, Google Scholar, and Cochrane Library using searching keywords “postoperative ischemic stroke, atrial fibrillation, stroke, cardiac surgery, brain ischemia, and heart surgery”. Direct and indirect comparisons were made using random-effect network meta-analysis.

**Results:**

16-studies were identified comprising of 132,208 patient, 64% male, median age > 63 years and follow-up > 1.5 years. Pooling the results from the random-effects model showed odds ratios associated with the risk of stroke of surgical processes (CABG) in patients with AF. The odds ratio OR = 1.1 (0.65–1.54, *P* < 0.001) and heterogeneity (I2 = 17%, *P* = 0.13) exposing higher risk of the stroke. Odds ratio (HR 1.5, 0.9–1.71) without heterogeneity showed greater risk of stroke after heart valve surgery in patients with AF. Study 8 didn’t show any risk of the stroke after left atrial appendage (LAA) clipping intervention, but the outcomes were biased. A pooled analysis showed odd ratio OR, 2 (1.7–2.1, *P* < 0.0001), without heterogeneity indicating higher stroke risk in general cardiac surgery. The patients undergone cardiac surgery from three studies with pooled analysis study-5 OR 2 (1.7–2.1, *P* > 0.001), study-6 OR 1.8 (1.7–1.9, *P* > 0.001), and study-14 OR 7.8 (6.2–8.1, *P* > 0.0001).

**Conclusion:**

The study clearly defines stroke outcomes when they are quantified, however, further research is required.

**Supplementary Information:**

The online version contains supplementary material available at 10.1186/s12872-025-04605-y.

## Introduction

Considering its association to ischemic stroke, atrial fibrillation (AF), the most prevalent persistent cardiac arrhythmia, affects millions of individuals worldwide (about 1% of the world population) and has significant sociomedical repercussions [1, 2]. It is major heart rhythm disruption and causes a significant amount of morbidity and death in the broader community. A poorly regulated or irregular heart beat causes its symptoms, and people with a history of AF are twice as likely to die from the condition [3]. Atherosclerosis patients with established chronic atrial fibrillation have a greater risk of stroke, longer hospital admissions, a higher chance of death, and worse functional outcomes than stroke patients without atrial fibrillation [4, 5]. New-onset postoperative atrial fibrillation (POAF) is the term used to describe the occurrence of atrial fibrillation (AF) in individuals who had previously had normal sinus rhythm (NSR) and had no prior history of AF following surgery. It is the most important type of secondary AF [6–11]. POAF is a frequent surgical complication that can occur in 10–63% of heart procedures (38-63% in valve surgeries or [Percutaneous coronary intervention (PCI), Transcatheter aortic-valve replacement (TAVR) left atrial appendage clipping surgery (LAAC), Left atrial appendage occlusion (LAAO)] and 10-33% in coronary artery bypass graft [CABG] surgeries) [12–14]. Although systemic anticoagulants-based therapy is quite effective, many patients find it difficult to maintain over time. As a result, there is a growing search for alternative approaches, particularly for those who are most at risk [15–21]. Given the data that shows the left atrial appendage (LAA) is the prime location of thrombus formation and consequent cardioembolic stroke in individuals with AF, nonpharmacological methods to separate the LAA from the systemic circulation are being developed [22–26]. More recently, several studies—though others were more cautious or equivocal in their conclusions—have linked AF to an increased risk of stroke over extended postoperative periods [27–32]. We hypothesized that the patient’s with AF undergone surgical interventions are correlated to an increased risk of strokes over long post-surgical periods. The goal of our study was to examine the risk of ischemic or haemorrhagic strokes in patients with a history of AF at least six months after surgery by conducting a systematic review and meta-analysis of current randomized controlled clinical trials (RCTs) and prospective studies. The outcomes discovered that AF is linked to a higher risk of stroke and death, both in the short and long term, as compared to populations without AF.

## Methodology and procedures

### Methods

**Fig. 1:**
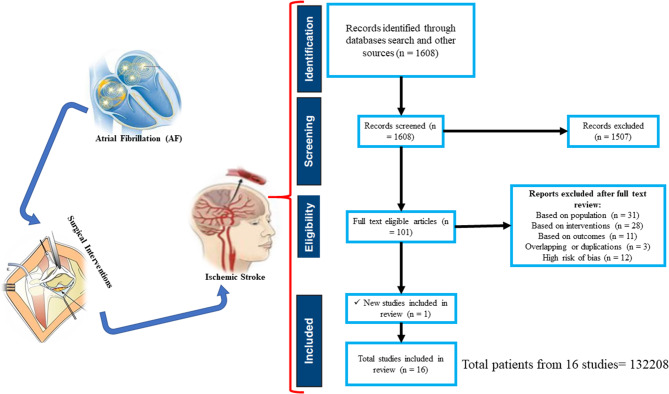
Preferred Reporting Items for Systematic Reviews and Meta Analyses (PRISMA) flowchart of review process to extract data for meta-analysis

The data and findings of this study are contained in the paper and its online supplementary information’s for the readers to reproduce the results or replicate the procedures. For data collection, a thorough search was made in PubMed, EMBASE, Science Direct, Google Scholar, and Cochrane Library using searching keywords “postoperative ischemic stroke, atrial fibrillation, stroke, cardiac surgery, brain ischemia, and heart surgery”. Studies only conducted on humans were included and all non-human studies were rejected. The studies reported in full text English language were considered. Furthermore, manual searches were made through all relevant data base for relevant publications [28, 33, 34]. The data are reported in accordance with the Preferred Reporting Items for Systematic Reviews and Meta-Analyses (PRISMA) guidelines for accomplishing the network meta-analysis (as given in Fig. 1).

### Selection of studies and data extraction

The authors independently searched the specified data based for literature to find out the relevant studies for meta-analysis. Full text articles were read and reviewed for making the decision to include or exclude. The studies were included or excluded based on the inclusion or exclusion criteria. The inclusion criteria were to have the following points; ① the patients might have present or past history of the AF, ② the patients might undergo for any surgical treatment for any cardio vascular diseases, ③ the patients must have age equal or above 18-years, ④ all studies included must be human data, ⑤ the sample size of the patients must be at least 50 patients, ⑥ the follow-up should be done for at least 6-months. The exclusion criteria were ① All non-human data, ② all non-surgical interventions ③ all strokes’ studies without AF, ④ AF without surgery, ⑤ surgery without AF, ⑥ where stroke incidences are not observed. We comprised investigations that gathered data from cohort studies either prospectively or retrospectively; patients underwent surgical procedures and had their baseline AF assessed; incident stroke and mortality were evaluated; and quantitative estimates of the multiple adjusted odds ratio (OR) or hazard ratio (HR) and 95% confidence interval (CI) for stroke and mortality related to perioperative or postoperative AF were reported. For randomized research, the Cochrane Collaboration risk of bias assessment instrument was utilized, and for nonrandomized studies, the Cochrane Robins-I scale [28]. The writers worked together to cross-check the data and reached a consensus when there were disagreements.

### Quality assessment

Next, we also assessed the quality of the eligible studies by preparing quality assessment checklist. The checklist was characterized as: the design of the study, comparable groups, outcomes, potential confounders (like age, sex, male/female, others), and documented follow-up. The studies were considered of good quality having 3 or more score, while below 3 were considered poor. This quality assessment was made in order to perform smooth and healthier review process to obtain the more reliable data.

### Types of surgical interventions

As various reports were found out with various surgical interventions for the treatment of AF or other CVS conditions. Therefore, we categorized the types of surgical interventions for the smooth analysis of data. The studies where patients with AF undergone coronary artery bypass graft (CABG) surgeries were group together. The valve surgeries like percutaneous coronary intervention (PCI), transcatheter aortic-valve replacement (TAVR), AF thoracic surgery, left atrial appendage clipping (LAAC), and left atrial appendage occlusion (LAAO) were grouped together for analytical assessments of the data. Those studies where only the general cardiac surgeries were mentioned without more information’s were grouped as cardiac surgical interventions. In two studies the interventions were cardiac versus non-cardiac surgery, and strokes rates were reported with prior discontinuation of warfarin after surgeries.

### Observed outcomes

Cerebrovascular events, including ischemic and haemorrhagic strokes, all-cause mortality, myocardial infarction, and a composite of haemorrhaging strokes were considered the favourable outcomes. Data were taken at each study’s most recent follow-up period as provided. The primary safety outcome was major bleeding as defined by the ISTH (International Society on Thrombosis and Haemostasis) definition, TIMI (Thrombolysis in Myocardial Infarction), BARC (Bleeding Academic Research Consortium), or GUSTO (Global Use of Strategies to Open Occluded Arteries).

### Data analysis

For outcomes, the pooled data were reported using the odds ratio and 95% CI. Direct and indirect comparisons as well as the synthesis of study impact sizes for every outcome were made possible using random-effect network meta-analysis and to explore the sources of heterogeneity and inconsistency (*I*^2^). P-values were two-sided, and the significance level was set at less than 0.05. The findings of interest were the early and long-term death rates as well as the risks of stroke among AF patients. The patients in the reference group did not have AF at baseline. We further divided the patient population into cardiac and noncardiac surgical types, and we contrasted the long-term outcomes odds ratio or HRs for strokes and death [28, 30].

## Results

After screening, we recognized 101 full length English language articles for further review process. 59 studies were excluded due to small population size or non-cardiac surgical interventions or not involving any surgery. 11 studies were excluded due to undesirable outcomes and 3 for reporting duplicate outcomes, while 12 for not having appropriate data on HRs/ORs or CIs. During this review process a new study was also identified and added for analysis. Totally 16 studies were identified that were comprising of total 132,208 patient, on average approximately 64% of the patients were male (reported in Figure S1).

The characteristics of the included studies are given in the Table 1, where the median age of the patients was found to be more than 63 years. The median follow-up after surgeries was noted to be more than 1.5 years. The total patients in the 16-studies were 147,929, after the exclusion of those patients who failed in follow-up or died due to other reasons than stroke the patients included in the meta-analysis remained 132,208.

**Table 1:** Base line characteristics of the included studies

Study	Total Patients (*n*)	Patients with AF (*n*)	Gender	Age (y)	Follow up (y)	References
Study 1	2236	2236	78.3% (male)	74.3 ± 8.3	2.0-3.7	Yasuda et al., 2019 [3]
Study 2	4614	4614	Random	54 ± 5	> 1	Lopes et al., 2019 [6]
Study 3	1403	1403	Random	74	1.0–2.0	Popma et al., 2019 [8]
Study 4	24,711	13,952	59.5% (male)	> 18	1	Gialdini et al., 2014 [22]
Study 5	150	150	60% (male)	52 ± 8.2	0.5–1.4	Wang et al., 2015 [33]
Study 6	69,202	69,202	56% (male)	45 ± 19.5	> 1	Kaatz et al., 2010 [32]
Study 7	26,046	26,046	51% (male)	≥ 67	1.3 to 1.5	Koshy et al., 2019 [9]
Study 8	3068	3068	53%(male)	18<	> 1	Lotfi et al., 2011 [12]
Study 9	382	382	male 73.3%	≥ 70	> 1	Barbieri et al., 2013 [24]
Study 10	161	161	males (77%)	72	8.5	Konstantino et al., 2016 [1]
Study 11	7145	2183	males (79%)	68.9 ± 7.7	9.8	Thorén et al., 2020 [14]
Study 12	186	186	male 63.3%	≥ 75	1	Ye et al., 2022 [31]
Study 13	1014	1014	males (57%)	75 ± 8	2	Kefer et al., 2015 [2]
Study 14	3474	3474	males (59%)	63 ± 7.3	> 1	Işık et al., 2021 [25]
Study 15	4060	4060	males (71.5%)	≥ 65	3.5 ± 1.3	Qureshi et al., 2016 [13]
Study 16	77	77	males (59%)	> 65	0.5–1.6	Healey et al., 2005 [7]

**Fig. 2:**
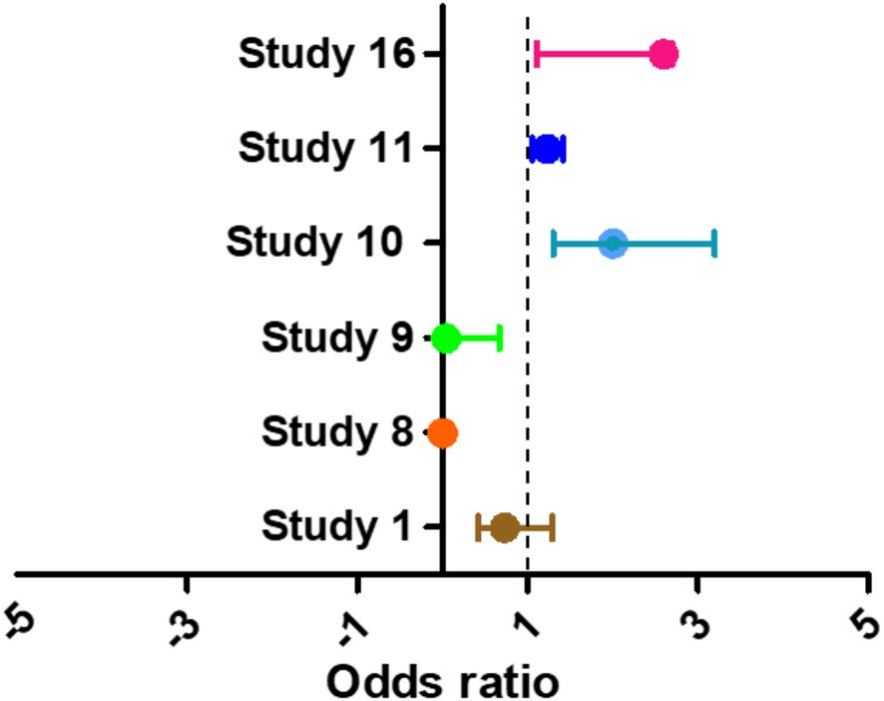
The forest plot Coronary-artery bypass grafting (CABG) patients for risk of stroke. OR 1.1 (0.65–1.54, *P* < 0.001), Heterogeneity (*P* = 0.13, *I*^2^ = 17%)

The pooled analysis (Odds ratio, 95% CI) are reported in Fig. 2, and the observed outcomes are given in Table 2 for the patients undergone CABG surgery having the positive history of the AF [1, 3, 7, 12, 14, 24]. The odds ratios are associated with the risk of stroke of surgical processes (CABG) in patients with AF. The odds ratio showed a significantly higher risk of the stroke in these patients having AF; OR = 1.1 (0.65–1.54, *P* < 0.001). The heterogeneity (*I*^2^ = 17%) was found insignificant (*P* = 0.13). The pooled analysis suggested a high risk of stroke in the patients undergoing CABG surgery with AF.

The total patients with AF and received CABG surgical intervention were 8107, who were successful in follow-up. The patients were observed for post-operative strokes and other cardiovascular events, while study 8 was based for POAF risks outcomes. Study 8 didn’t show any case of stroke after CABG surgery instead showed cases of POAF occurrence in these patients. Although this study was included with poor quality assessments because of fulfilling the designed inclusion criteria and parameters [21]. The authors discussed the study and added. The odds ratio of the studies for stroke risk and observed outcomes are also given in the Table 2. The analysis revealed the risks of occurrence of stroke after CABG intervention.

**Table 2:** Coronary-artery bypass grafting (CABG) patients with AF, observed outcomes and hazard ratio

Reports	(*n*)	Surgical interventions	Observed outcomes	HR (95%, CI)
Study 1	2236	CABG	Stroke, Systemic embolism, MI, Unstable angina	0.73 (0.42–1.29)
Study 8	3068	CABG	POAF	-
Study 9	382	CABG	Stroke	0.04 (0.01–0.67)
Study 10	161	CABG	Stroke, Cerebrovascular accident (CVA), or death.	1.6 (1.3–2.1)
Study 11	2183	CABG	POAF	1.23 (1.06–1.42)
Study 16	77	CABG	Postoperative Stroke	2.6 (1.11–2.7)

**Fig. 3:**
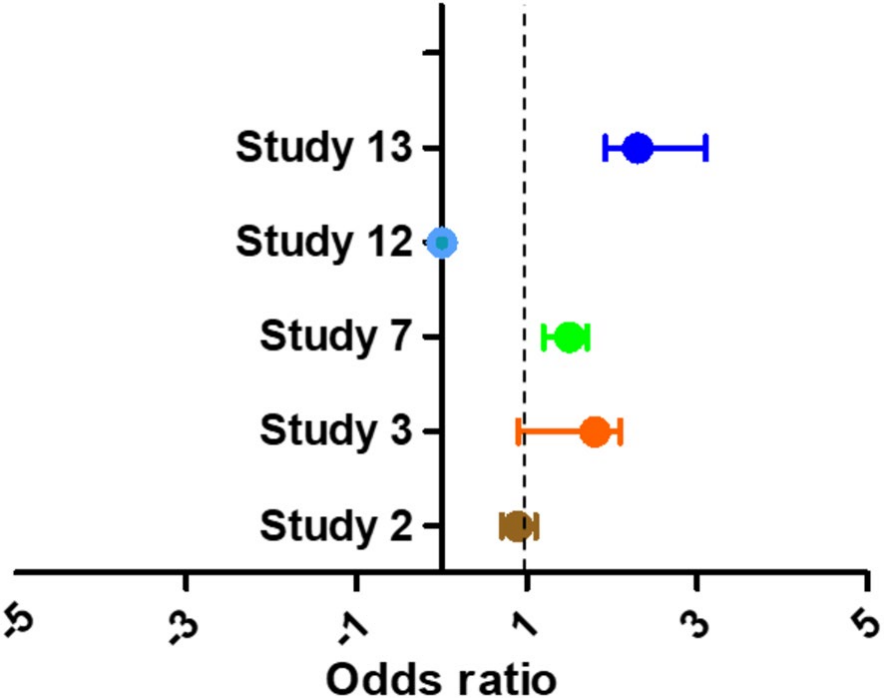
The forest plot heart valves surgeries patients with AF for the risk of stroke. OR 1.5 (0.9–1.71, *P* < 0.001), Heterogeneity (*P* = 0.67, *I*^2^ = 0%)

Even though stroke is one of the most feared consequences of cardiac surgery, the risk of stroke during or after heart valve surgery is minimal. Some of the risk factors that can increase the risk of stroke after heart valve surgery include carotid artery stenosis greater than 50%, redo heart surgery, valve surgery, mortality risk, advanced age, and previous history of stroke. It is noteworthy that there is an increased risk of operational death for patients who suffer from perioperative stroke following heart valve surgery. 5 studies were included for meta-analysis in this study to pool the analysis for risk of stroke after heart valve surgery (Fig. 3) [2, 6, 8, 9, 31]. The level of odds ratio (HR 1.5, 0.9–1.71) showed greater risk of stroke after heart valve surgery in patients with AF with P-valve less than 0.001 showing remarkable significance. The heterogeneity was not found with higher value (0.63) of P-value. Study 8 didn’t show any risk of the stroke after LAAC intervention.

The interventions for surgeries in these studies were PCI, TAVR, AFT, LAAC, and LAAO, and the respective observational outcomes have been reported in the Table 3. The study 13 focused observations were the efficiency of LAAO in patients already suffering from the chronic kidney diseases (CKD). The study represented a high risk of stroke in the patients with AF and suffered from the CKD. 33,263 patients with AF were enrolled with successful follow up in these publications.

**Table 3:** Patients with AF undergone value surgery, observed outcomes and hazard ratio

Reports	(*n*)	Surgical interventions	Observed outcome	HR (95%, CI)
Study 2	4614	PCI	Composite of ischemic events	0.89 (0.71–1.11)
Study 3	1403	TAVR	Disabling stroke at 24 months	1.8 (0.9–2.1)
Study 7	26,046	AFT	Stroke risk	1.5 (1.2–1.71)
Study 12	186	LAAC	Stroke, Systemic embolism, MI	-
Study 13	1014	LAAO	Efficiency of LAAO in patients with CKD.	2.3 (1.92–3.1)

**Fig. 4:**
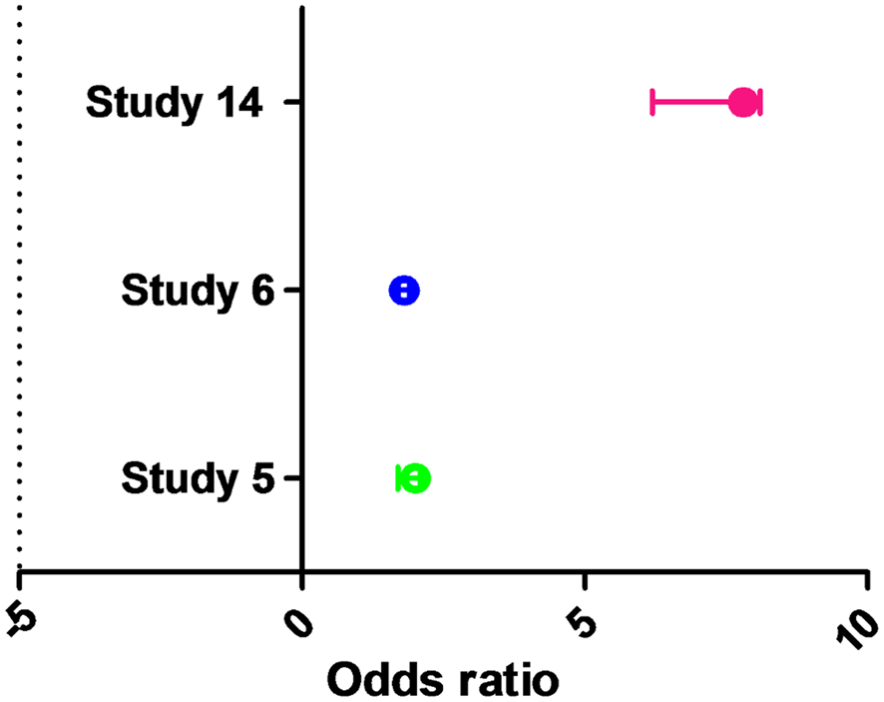
The forest plot of cardiac surgery patients with AF for the risk of stroke. OR, 2 (1.7–2.1, *P* < 0.0001), Heterogeneity (*P* = 0.77, *I*^2^ = 0%)

Operative death rates were greater in individuals with perioperative stroke, early stroke, and delayed stroke than in patients without stroke with relative history of the AF. In addition, the incidence rate of late death was greater in patients with early stroke than in patients with delayed stroke. The incidence of stroke after cardiac surgery ranges from 0.8 to 5.2%. The burden of stroke has been a major limitation for surgery in landmark trials comparing outcomes of coronary artery bypass graft and percutaneous coronary intervention. The studies with cardiac surgeries (not mentioned with specific surgical procedure, rather carried single or multiple cardiac surgeries) were include together to pool the analysis for risk of stroke [25, 32, 33]. A pooled analysis showed odd ratio OR, 2 (1.7–2.1, *P* < 0.0001), without any heterogeneity (P 0.77) indicating the correlation of higher stroke risk in patients with AF after cardiac surgery. The forest plot for three studies is reported in Fig. 4. Following heart surgery, perioperative atrial fibrillation has been linked to a higher risk of stroke. New-onset perioperative atrial fibrillation was linked to a longer-term and greater risk of stroke, according to a meta-analysis.

A total of 72,826 AF patients undergone cardiac surgery were included from three studies with pooled analysis study 5 OR 2 (1.7–2.1, *P* > 0.001), study 6 OR 1.8 (1.7–1.9, *P* > 0.001), and study 14 OR 7.8 (6.2–8.1, *P* > 0.0001) as reported in Table 4. The observed outcomes were post-operative stroke (ischemic and haemorrhagic strokes). In patients having heart surgery, postoperative atrial fibrillation (AF) is linked to a higher risk of stroke and a higher death rate. It is crucial to remember that patients who have perioperative stroke following cardiac surgery are more likely to die during the procedure, even if the risk of stroke following cardiac surgery is minimal [28]. Age, prior stroke experience, and carotid artery stenosis are some of the variables that may increase the risk of stroke following heart surgery.

**Table 4:** Patients with AF undergone valve surgery, observed outcomes and hazard ratio

Reports	(*n*)	Surgical Interventions	Observed outcomes	HR (95%, CI)
Study 5	150	Cardiac surgery	Postoperative Stroke and others	2 (1.7–2.1)
Study 6	69,202	Cardiac surgery	Postoperative Stroke with AF	1.8 (1.7–1.9)
Study 14	3474	Cardiac surgery	Pathophysiological mechanisms of acute ischemic stroke (AIS) that develop after cardiac surgery.	7.8 (6.2–8.1)

Between 3 and 30% of noncardiac procedures result in atrial fibrillation (AF), with a greater prevalence following thoracic surgeries. Where the incidence of stroke after cardiac surgery has been reported in follow up studies, more higher strokes rates were found after cardiac surgeries rather than noncardiac surgeries. Although it is known, the risk of stroke following warfarin withdrawal for surgical operations is not well understood. Nonetheless, some information on the subject is provided by the items that follow. The risk of an ischemic stroke increases when high-risk atrial fibrillation patients stop taking warfarin for surgical operations. Participants who stopped taking warfarin due to surgery had an increased incidence of ischemic stroke. Even after accounting for possible confounders, stopping warfarin was linked to a higher risk of ischemic stroke. For ischemic stroke, the population-attributable risk of stopping warfarin due to surgery was calculated to be 23.1%. Recent evidence indicates that stopping warfarin in patients with vascular indications increases the risk of arterial thromboembolism more than assumptions used in mathematical modelling, particularly in the early postoperative phase. Here, two studies regarding warfarin discontinuation before surgical interventions and cardiac vs. non-cardiac surgical interventions were included for analysis of stroke rates (Supporting Table 1). 18,014 patients from the two studies were included to pool the analysis [13, 22]. The odd ratio was OR 15.2 (23.1–30.9, *P* < 0.05) and OR 0.79 (0.81–0.82, 0.001) for risk of stroke in the study 15 and study 4, respectively. The heterogeneity levels were (*I*^2^ = 50%, *P* = 0.04) the heterogeneity was found significant, and (*I*^2^ = 13%, *P* = 0.81) incognisant levels of heterogeneity were recorded. These analyses also revealed an increased risk of stroke in the patients with AF undergone surgeries.

## Discussion

**Fig. 5:**
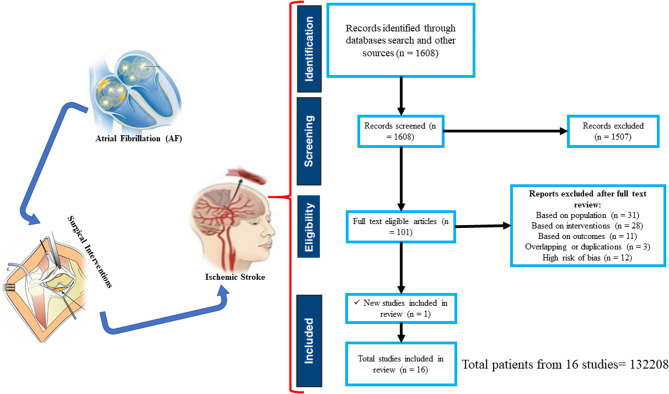
Clinical perspective of the study

Unambiguously, AF appears to be associated with perioperative mortality, perioperative stroke, perioperative myocardial infarction, perioperative acute renal failure, and long-term mortality, long-term stroke, longstanding persistent AF, as well as hospital length of stay and intensive care unit length of stay (Fig. 5). Our analysis suggests that AF in patients following cardiac surgery is associated with an increased occurrence of most short- and long-term cardiovascular adverse events [1, 4, 5, 12, 29]. It is unclear whether AF had a role in the pathophysiology of the related events or if it was only a marker of elevated cardiovascular risk, given our data do not establish causality. According to recent evidence, a preexisting arrhythmogenic substrate that determines who would develop AF existed before surgery. It may be able to explain the incidence of other cardiovascular events as well as the long-term recurrence rate of strokes after AF. Susceptible atrial substrates are known to be overlaid by variables such inflammation, myocardial ischemia, and autonomic nervous system activity, which leaves the atrium open to AF development and maintenance. Furthermore, individuals who already have atrial fibrosis may be more likely to develop AF, which may affect when cardiac procedures should be performed [1, 4, 22, 29, 5, 12, 15, 16, 18–21].

This systematic review and network meta-analysis suggests that AF patients undergoing cardiac surgical interventions are not only associated with the higher risk of major bleeding, but all-cause mortality. CABG, PCI, TAVR, AFT, LAAC, LAAO, DOAC, and AF thoracic surgery are associated with high rates of stroke. Although patients receiving anticoagulants showed comparably reduced incidence of the stroke with AF [35]. Expert opinion statements and recommendations that were recently released advise treating patients with atrial fibrillation (AF) after surgery by combining a single anticoagulant and a single antiplatelet drug (P2Y12 inhibitor) [6, 7, 21–25]. The findings shown in this meta-analysis support and reinforced these suggestions, favouring the AF patients who underwent surgery with or without medication. It’s interesting to note that stopping anticoagulants before to heart surgery was associated with a greater risk of ischemic stroke, according to a signal that suggests a link between the two. Conversely, taking anticoagulants was linked to a lower risk of significant bleeding and stroke, two factors that are crucial for patients with AF. Furthermore, our findings imply that individuals who have had surgery and AF are more likely to experience recurrent coronary events and strokes [2, 9–11, 26]. The findings of this meta-analysis strongly suggest a correlation between a higher long-term risk of stroke and AF that develops following either isolated cardiac bypass surgery or combined bypass surgery and/or valve replacement surgery. In the pooled analysis for risk of stroke, the degree of heterogeneity in the study outcomes was modest. Differences in the quality and the characteristics of the population were partially explained by research related to the heterogeneity’s explanation [2, 4, 9–11, 26, 31, 32]. A higher number of men were typically related with a greater risk of AF-associated stroke in studies, but this was a minor impact and a weakly significant interaction. Secondly, the number of follow up patients were also higher compared to women. According to a recent study, there was no discernible correlation between sex and risk over this shorter time frame for AF-related stroke.

One meta-analysis of patients who had undergone CABG revealed a similarly increased NOAF-associated long-term mortality risk (odds ratio 2.19, 95% CI, 2.14–3.08) at 1-year follow-up. It is thought-provoking to determine the impact of the prevalence of continuous anticoagulants on the risk of AF-associated stroke following heart surgery because this information was not stated clearly in other publications, but one study included in this analysis did [13–15, 28]. When compared to retrospective and better-quality research, both prospective studies and studies of lower quality—two categories of studies that mainly overlapped—showed a substantially greater risk of AF-associated stroke. This might be the case because studies of lower quality tended to define stroke more loosely, which made it more probable that more cases of this event would be included. This study has limitations that are typical of all meta-analyses. It might not be fitting to systematically combine studies when the effect variation due to heterogeneity is high, and it might not be appropriate to compare studies on populations with different outcomes, adjustment variables, or inclusion/exclusion criteria even when the effect variation due to heterogeneity is statistically low. The studies we found showed a considerable amount of impact variation due to heterogeneity, which might be partially explained by subgroup analysis on the surgical population under investigation. This might have led to an overestimation of the AF-associated stroke data in some studies by including patients with pre-existing AF. Many of the included studies poorly defined the stroke outcomes, which may have introduced a detection bias. Most did not distinguish between ischemic and haemorrhagic outcomes [7]. Furthermore, no studies classified ischemic strokes by cause or determined the timing of AF recurrences and stroke.

## Conclusion

Following CABG or cardiac valve surgery, AF is linked to a higher risk of stroke and death, both in the short and long term, as compared to populations without AF. To ascertain if anticoagulation lowers risk or whether this elevated risk is mediated by AF, more investigation is necessary. To determine if anticoagulation is necessary for AF patients, longer-term monitoring may be necessary. Future research in this area needs to clearly define stroke outcomes when they are quantified.

## Limitations and future research directions

We focused on the quantification of stroke outcomes with atrial fibrillation and association between atrial fibrillation and surgery with stroke was valued via this systematic review and meta-analysis. However, there are some potential limitations of this study including heterogeneity in the populations, variations in surgery, no detailed description of stroke subtypes, lack of individual patient data, and bias in publications reported. The future research may focus on individualized patient meta-analysis to allow for more detailed analyses and adjustment for potential confounders. It’s also noteworthy to perform subgroup analyses based on characteristics of the atrial fibrillation, such as its duration, frequency, and burden, to better understand the relationship between atrial fibrillation and stroke incidence. Moreover, continuous observations and conducting separate studies for patients with longstanding atrial fibrillation and those with paroxysmal atrial fibrillation might be helpful to minimize heterogeneity.

## Electronic supplementary material

Below is the link to the electronic supplementary material.

Supplementary Material 1

## Data Availability

The data will be available from the corresponding author upon reasonable request.

## Abbreviations

AF: Atrial fibrillation
POAF: New-onset postoperative atrial fibrillation
NSR: Normal sinus rhythm
PCI: Percutaneous coronary intervention
TAVR: Transcatheter aortic-valve replacement
LAAO: Left atrial appendage occlusion
LAA: Left atrial appendage
RCTs: Randomized controlled clinical trials
PRISMA: Preferred Reporting Items for Systematic Reviews and Meta-Analyses
CABG: Coronary artery bypass graft
LAAC: LAA clipping
ISTH: International Society on Thrombosis and Haemostasis
TIMI: Thrombolysis in Myocardial Infarction
BARC: Bleeding Academic Research Consortium
GUSTO: Global Use of Strategies to Open Occluded Arteries

## References

[1] Konstantino Y, Zelnik Yovel D, Friger MD, Sahar G, Knyazer B, Amit G. Postoperative atrial fibrillation following coronary artery bypass graft surgery predicts Long-Term atrial fibrillation and stroke. Isr Med Association Journal: IMAJ. 2016;18(12):744–8. 28457078

[2] Kefer J, et al. Impact of chronic kidney disease on left atrial appendage occlusion for stroke prevention in patients with atrial fibrillation. Int J Cardiol. 2016;207:335–40. 26820363 10.1016/j.ijcard.2016.01.003

[3] Yasuda S, et al. Antithrombotic therapy for atrial fibrillation with stable coronary disease. N Engl J Med. 2019;381(12):1103–13. 31475793 10.1056/NEJMoa1904143

[4] Caldonazo T, et al. Atrial fibrillation after cardiac surgery: A systematic review and meta-analysis. J Thorac Cardiovasc Surg. 2023;165(1):94–e103. 33952399 10.1016/j.jtcvs.2021.03.077

[5] Lin MH, Kamel H, Singer DE, Wu YL, Lee M, Ovbiagele B. Perioperative/Postoperative atrial fibrillation and risk of subsequent stroke and/or mortality: A Meta-Analysis. Stroke. 2019;50(6):1364–71. 31043148 10.1161/STROKEAHA.118.023921

[6] Lopes RD, et al. Antithrombotic therapy after acute coronary syndrome or PCI in atrial fibrillation. N Engl J Med. 2019;380(16):1509–24. 30883055 10.1056/NEJMoa1817083

[7] Healey JS, et al. Left atrial appendage occlusion study (LAAOS): results of a randomized controlled pilot study of left atrial appendage occlusion during coronary bypass surgery in patients at risk for stroke. Am Heart J. 2005;150(2):288–93. 16086933 10.1016/j.ahj.2004.09.054

[8] Popma JJ, et al. Transcatheter Aortic-Valve replacement with a Self-Expanding valve in Low-Risk patients. N Engl J Med. 2019;380(18):1706–15. 30883053 10.1056/NEJMoa1816885

[9] Koshy AN et al. Postoperative atrial fibrillation following noncardiac surgery increases risk of stroke. Am J Med. 2020;133(3):311–322.e5. 10.1016/j.amjmed.2019.07.057 31473150

[10] Wang C, et al. Risk model of prolonged intensive care unit stay in Chinese patients undergoing heart valve surgery. Heart Lung Circulation. 2012;21(11):715–24. 22898595 10.1016/j.hlc.2012.06.018

[11] Saw J, Bennell MC, Singh SM, Wijeysundera HC. Cost-Effectiveness of left atrial appendage closure for stroke prevention in atrial fibrillation patients with contraindications to anticoagulation. Can J Cardiol. 2016;32(11):1355. 10.1016/j.cjca.2016.02.056 27432692

[12] Lotfi A, Wartak S, Sethi P, Garb J, Giugliano GR. Postoperative atrial fibrillation is not associated with an increase risk of stroke or the type and number of grafts: A single-center retrospective analysis. Clin Cardiol. 2011;34(12):787–90. 22120735 10.1002/clc.21001 PMC6652536

[13] Qureshi AI, Jahangir N, Malik AA, Afzal MR, Orfi F, Suri MFK. Risk of ischemic stroke in high risk atrial fibrillation patients during periods of warfarin discontinuation for surgical procedures. Cerebrovasc Dis. 2016;42:5–6. 10.1159/000446406 27322535

[14] Thorén E, Wernroth ML, Christersson C, Grinnemo KH, Jidéus L, Ståhle E. Compared with matched controls, patients with postoperative atrial fibrillation (POAF) have increased long-term AF after CABG, and POAF is further associated with increased ischemic stroke, heart failure and mortality even after adjustment for AF. Clin Res Cardiol. 2020;109(10):1232–42. 32036429 10.1007/s00392-020-01614-z PMC7515855

[15] Hart RG. Stroke prevention in atrial fibrillation. Antithrombotic Therapy Chronic Heart Disease 2000;2(1):51–5. 10.1007/s11886-000-0025-2. 10.1007/s11886-000-0025-2 10980872

[16] Guo Y, Lip GYH, Apostolakis S. Bleeding risk assessment in patients with atrial fibrillation who are taking oral anticoagulants bleeding risk assessment in patients with atrial fibrillation who are taking oral anticoagulants. Hosp Pract. 2015;8331(November):70–71. 10.3810/hp.2013.02.1012 23466969

[17] Hacke W, et al. Rivaroxaban versus warfarin in nonvalvular atrial fibrillation. N Engl J Med. 2011;365(10):883–91. 21830957 10.1056/NEJMoa1009638

[18] Gottdiener JS, et al. Predictors of congestive heart failure in the elderly: the cardiovascular health study. J Am Coll Cardiol. 2000;35(6):1628–37. 10807470 10.1016/s0735-1097(00)00582-9

[19] Therapy P, Cardioversion E, Mcnamara RL, Tamariz LJ, Segal JB, Bass EB. Clinical guidelines management of atrial fibrillation: review of the evidence for the. Ann Intern Med. 2003;139:1018–33. 14678922 10.7326/0003-4819-139-12-200312160-00012

[20] Education H, Outcomes P. Prevalence and incidence of hypertension from 1995 to 2005: a population-based study. CMAJ. 2008;178(11):1429–35. 18490638 10.1503/cmaj.071283 PMC2374870

[21] Torregrosa M, Ga J. Relation of the HAS-BLED bleeding risk score to major bleeding, cardiovascular events, and mortality in anticoagulated patients with atrial fibrillation. Circ Arrhythm Electrophysiol. 2012;5(2):312–8. 10.1161/CIRCEP.111.967000 22319005

[22] Gialdini G, et al. Perioperative atrial fibrillation and the long-term risk of ischemic stroke. JAMA-Journal Am Med Association. 2014;312(6):616–22. 10.1001/jama.2014.9143 PMC4277813 25117130

[23] Trial ATR, et al. Concomitant use of antiplatelet therapy with Dabigatran or warfarin in the randomized evaluation of Long-Term anticoagulation therapy (RE-LY) trial. Circulation. 2012;127(5):634–40. 10.1161/CIRCULATIONAHA.112.115386 23271794

[24] Barbieri LR, et al. Incidência de Acidente vascular Encefálico e insuficiência renal Aguda Em Pacientes com fibrilação atrial no pós-operatório de cirurgia de revascularização do Miocárdio. Brazilian J Cardiovasc Surg. 2013;28(4):442–8.

[25] Işik M, Kozak HH, Görmüş N. Relationship between cardiac surgery and acute ischemic stroke: an examination in terms of clinical, radiological, and functional outcomes and possible pathophysiological mechanisms. Heart Surg Forum. 2021;24(4):E713–23. 34473018 10.1532/hsf.4007

[26] Knijnik L, et al. Prevention of stroke in atrial fibrillation after coronary stenting: systematic review and network Meta-Analysis. Stroke. 2019;50(8):2125–32. 31303150 10.1161/STROKEAHA.119.026078

[27] Santini M, Loiaconi V, Tocco MP, Mele F, Pandozi C. Feasibility and efficacy of minimally invasive stand-alone surgical ablation of atrial fibrillation. A single-center experience. J Interventional Cardiac Electrophysiol. 2012;34(1):79–87. 10.1007/s10840-011-9650-5 PMC3342490 22231157

[28] D’Ascenzo F, et al. Which are the most reliable predictors of recurrence of atrial fibrillation after transcatheter ablation? A meta-analysis. Int J Cardiol. 2013;167(5):1984–9. 22626840 10.1016/j.ijcard.2012.05.008

[29] da Silva FM, et al. Influence of external temporary biatrial pacing on the prevention of atrial fibrillation after coronary artery bypass without extracorporeal circulation. Arquivos Brasileiros De Cardiologia. 2008;90(2):80–5. 18392378

[30] Megens MR, Churilov L, Thijs V. New-Onset atrial fibrillation after coronary artery bypass graft and Long-Term risk of stroke: A Meta-Analysis. J Am Heart Association. 2017;6(12). 10.1161/JAHA.117.007558 PMC5779055 29273637

[31] Ye C et al. Stroke prevention of thoracoscopic left atrial appendage clipping in patients with non-valvular atrial fibrillation at high risk of stroke and bleeding: study protocol for a non-randomised controlled clinical trial. BMJ Open. 2022;12(10). 10.1136/bmjopen-2022-063931 PMC9621168 36307161

[32] Kaatz S, Douketis JD, Zhou H, Gage BF, White RH. Risk of stroke after surgery in patients with and without chronic atrial fibrillation. J Thromb Haemost. 2010;8(5):884–90. 20096001 10.1111/j.1538-7836.2010.03781.x

[33] Wang W, Mei YQ, Yuan XH, Feng XD. Clinical efficacy of epicardial application of drug-releasing hydrogels to prevent postoperative atrial fibrillation. J Thorac Cardiovasc Surg. 2016;151(1):80–5. 26254755 10.1016/j.jtcvs.2015.06.061

[34] Reddy VY, et al. Percutaneous left atrial appendage closure for stroke prophylaxis in patients with atrial fibrillation 2.3-year follow-up of the PROTECT AF (Watchman left atrial appendage system for embolic protection in patients with atrial fibrillation) trial. Circulation. 2013;127(6):720–9. 23325525 10.1161/CIRCULATIONAHA.112.114389

[35] Mohanty S. Best anticoagulation strategy with and without appendage occlusion for stroke-prophylaxis in postablation atrial fibrillation patients with cardiac amyloidosis. J Cardiovasc Electrophysiolvol. 2024;35:1422–8. 10.1111/jce.16308 38751010

