# Supplementary material for: Incidence of stroke in patients with atrial fibrillation undergoing surgical treatment: a meta-analysis
Source: BMC Cardiovasc Disord. 2025 Mar 29;25:233. doi: 10.1186/s12872-025-04605-y (PMC11954250; doi:10.1186/s12872-025-04605-y)
Supplement: Supplementary file 1 — Supplementary Material 1 [file 12872_2025_4605_MOESM1_ESM.docx]

**Supporting Information’s**

Meta-analysis

**Incidence of Stroke in Patients with Atrial Fibrillation Undergoing Surgical Treatment: A Meta-Analysis**

Deqing Lin^#^，Yongbo Cheng^#^, Sanjiu Yu, Xin Liu, Chaojun Yan, Wei Cheng^*^

Department of Cardiac Surgery, Southwest Hospital, Third Military Medical University (Army Medical University), Chongqing, 400038, P.R. China.

^#^These authors contributed equally

^*^Correspondence to: Wei Cheng, Department of Cardiac Surgery, Southwest Hospital, Third Military Medical University (Army Medical University), Chongqing, 400038, P.R. China. ([chw_yj@163.com](mailto:chw_yj@163.com); [chw_yj@hotmail.com](mailto:chw_yj@hotmail.com))


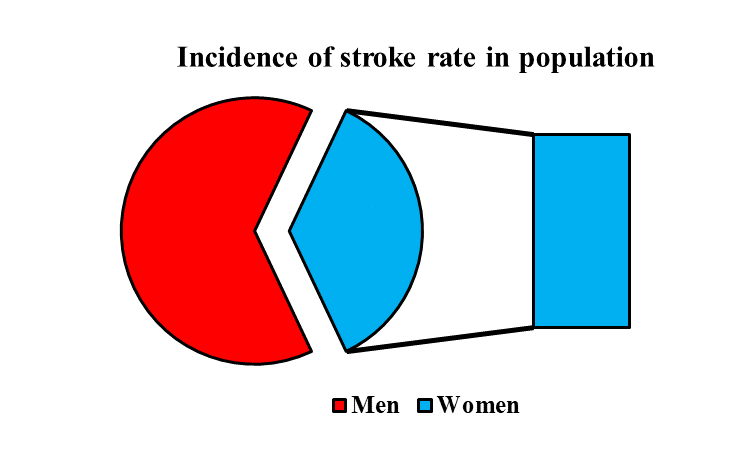


**Supporting Figure S1**. Stroke rates in men/women populations world-wide.

**Supporting Table 1.** Outcomes and HR of study 15 and study 4

| **Reports** | **(n)** | **Surgical interventions** | **Observed outcomes** | **HR (95%, CI)** |
| --- | --- | --- | --- | --- |
| Study 15 | 4060 | Warfarin discontinuation for surgical procedures | atrial fibrillation plus at least one other risk factor for stroke or death | 15.2  23.1  30.9 |
| Study 4 | 13952 | Perioperative atrial fibrillation after noncardiac vs cardiac surgery | Stroke | 0.79  0.81  0.82 |
